# Cuproptosis Depicts Immunophenotype and Predicts Immunotherapy Response in Lung Adenocarcinoma

**DOI:** 10.3390/jpm13030482

**Published:** 2023-03-08

**Authors:** Wolong Zhou, Yuanda Cheng, Linfeng Li, Heng Zhang, Xizhe Li, Ruimin Chang, Xiaoxiong Xiao, Liqing Lu, Bin Yi, Yang Gao, Chunfang Zhang, Junjie Zhang

**Affiliations:** 1 Department of Thoracic Surgery, Xiangya Hospital, Central South University, Changsha 410008, China; 2 Xiangya Lung Cancer Center, Xiangya Hospital, Central South University, Changsha 410008, China; 3 National Clinical Research Center for Geriatric Disorders, Xiangya Hospital, Central South University, Changsha 410008, China; 4 Hunan Key Laboratory of Molecular Precision Medicine, Xiangya Hospital, Central South University, Changsha 410008, China

**Keywords:** cuproptosis, experimental validation, lung adenocarcinoma, immunophenotype, immunotherapy response

## Abstract

Background: Although significant progress has been made in immunotherapy for lung adenocarcinoma (LUAD), there is an urgent need to identify effective indicators to screen patients who are suitable for immunotherapy. Systematically investigating the cuproptosis-related genes (CRGs) in LUAD may provide new ideas for patients’ immunotherapy stratification. Method: We comprehensively analyzed the landscape of 12 CRGs in a merged TCGA and GEO LUAD cohort. We investigated the associations between tumor microenvironment and immunophenotypes. We utilized a risk score to predict the prognosis and immunotherapy response for an individual patient. Additionally, we conducted CCK-8 experiments to evaluate the impact of DLGAP5 knockdown on A549 cell proliferation. Result: We utilized an integrative approach to analyze 12 CRGs and differentially expressed genes (DEGs) in LUAD samples, resulting in the identification of two distinct CRG clusters and two gene clusters. Based on these clusters, we generated immunophenotypes and observed that the inflamed phenotype had the most abundant immune infiltrations, while the desert phenotype showed the poorest immune infiltrations. We then developed a risk score model for individual patient prognosis and immunotherapy response prediction. Patients in the low-risk group had higher immune scores and ESTIMATE scores, indicating an active immune state with richer immune cell infiltrations and higher expression of immune checkpoint genes. Moreover, the low-risk group exhibited better immunotherapy response according to IPS, TIDE scores, and Imvigor210 cohort validation results. In addition, our in vitro wet experiments demonstrated that DLGAP5 knockdown could suppress the cell proliferation of A549. Conclusion: Novel cuproptosis molecular patterns reflected the distinct immunophenotypes in LUAD patients. The risk model might pave the way to stratify patients suitable for immunotherapy and predict immunotherapy response.

## 1. Background

Lung cancer is a prevalent malignant tumor worldwide, with approximately 2,206,771 newly diagnosed cases and 1,796,144 deaths reported in the latest statistics [1]. Among all lung cancer cases, lung adenocarcinoma (LUAD) is the major histological subtype, accounting for 40–50% [2]. Notably, significant advances have been made in immunotherapy for LUAD, resulting in sustained responses [2,3]. However, this effect is only observed in a small portion of LUAD patients. The complex landscape of molecular heterogeneity at the genomic and immunological levels makes it challenging to predict the immunotherapy response in LUAD patients [4]. In addition, inherent resistance to immunotherapy in some patients poses new challenges for clinicians.

The tumor microenvironment (TME) is gaining increased attention as a potential target for novel therapeutic strategies and a predictor of response to immune checkpoint inhibitors (ICIs) [4]. TME is a complex mixture of cells and factors, including cancer cells, infiltrating immune cells, stromal cells, signaling mediators, and extracellular matrix proteins [5]. Studies have shown that the presence of a chronic inflammatory TME in LUAD can disrupt immune cell differentiation, leading to an imbalance of antitumor activity that favors tumor evasion and resistance to ICIs [6]. Therefore, a comprehensive analysis of TME may aid clinicians in identifying effective immunotherapy-response indicators and designing personalized therapy plans for individual patients.

Cuproptosis is a recently discovered type of cell death triggered by an accumulation of excess copper ions within cells [7]. Ferredoxin 1 (FDX1) plays a critical role in this process by reducing Cu(II) to Cu(I) in mitochondria. FDX1 also promotes the lipoylation of dihydrolipoamide S-acetyltransferase (DLAT), which is involved in the formation of the pyruvate dehydrogenase (PDH) complex and the mitochondrial tricarboxylic acid (TCA) cycle [8]. When Cu(I) binds to lipoylated DLAT, it causes its oligomerization. FDX1 also promotes the synthesis of Fe-S cluster proteins, which are essential components of lipoic acid synthetase (LIAS) and electron transport chain (ETC), both of which are key enzymes involved in DLAT lipoylation [9]. However, Cu(I) can inhibit this process, leading to abnormal thioredoxin oligomerization and the loss of Fe-S clusters. These events ultimately result in proteotoxic stress response and cuproptosis [7]. Understanding the mechanisms of cuproptosis and its regulation may provide new therapeutic opportunities for various diseases and cancers.

Recent research has established a link between alterations in intracellular copper levels and the development and progression of LUAD [7]. Moreover, intracellular copper has been found to regulate the TME and facilitate PD-L1-mediated immune evasion through multiple signaling pathways [8]. Despite this, our understanding of the relationship between cuproptosis-related genes (CRGs) and TME, as well as immunotherapy in LUAD, remains limited. In this study, we investigated the correlations between 12 CRGs and prognosis, a novel molecular pattern, TME infiltration characteristics, immunophenotype, and the efficacy of precision immunotherapy. Our findings provide valuable insights for predicting LUAD prognosis and immunotherapy response, thereby enabling clinicians to accurately stratify patients for immunotherapy.

## 2. Methods

### 2.1. Multiomics Data Acquisition and Processing

**Figure 1:**
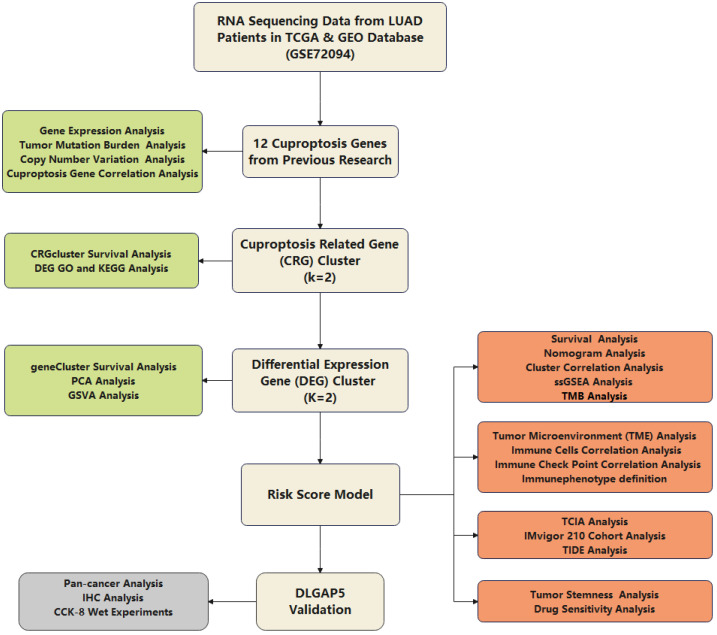
Flowchart of the present study. The light-yellow boxes indicate the main axis of the study design. The light-green boxes indicate the detailed analysis of 12 cuproptosis genes, CRGclusters, and geneClusters. The orange boxes indicate the detailed analysis between risk score and variables. The grey box indicates the detailed analysis of DLGAP5 from different aspects.

The flowchart of the present study is shown in Figure 1. In total, 12 CRGs, including ATPase copper transporting beta (ATP7B), solute carrier family 31 member 1 (SLC31A1), FDX1, LIAS, lipoyltransferase 1 (LIPT1), dihydrolipoamide dehydrogenase (DLD), DLAT, pyruvate dehydrogenase E1 subunit alpha 1 (PDHA1), pyruvate dehydrogenase E1 subunit beta (PDHB), metal regulatory transcription factor 1 (MTF1), glutaminase (GLS), cyclin-dependent kinase inhibitor 2A (CDKN2A), were mainly retrieved from a previous study [7]. We obtained the RNA-sequencing data and clinical information of LUAD patients from the TCGA (https://portal.gdc.cancer.gov/, accessed on 18 June 2022), [8], and GEO (https://www.ncbi.nlm.nih.gov/geo/, accessed on 18 June 2022, GSE72094) databases [9]. The two datasets were merged using the combat algorithm, and batch effects were minimized with the R package sva. A total of 958 LUAD samples, including 516 from the TCGA-LUAD cohort and 442 from GSE72094, were included in the study. Furthermore, mutation data for LUAD patients was obtained from the TCGA database, and copy number variation (CNV) data was obtained from UCSC Xena (UCSC Xena (xenabrowser.net)) [10]. Information on 59 normal samples was also collected from TCGA. The protein expression immunohistochemistry (IHC) images of CRGs in clinical specimens of LUAD patients were obtained from The Human Protein Atlas (HPA) (https://www.proteinatlas.org/, accessed on 18 June 2022) [11].

### 2.2. Consensus Clustering Analysis of CRGs

We used the R package ConsensusClusterPlus to conduct unsupervised consensus clustering analysis in order to identify distinct molecular patterns of cuproptosis-related genes in LUAD. ConsensusClusterPlus is one useful and popular method that allows for more precise decisions in unsupervised class discovery [12]. The most suitable clustering number was figured out based on the tendency and smoothness of the cumulative distribution function (CDF). We used the R package statistics to perform principal component analysis (PCA) to assess the effectiveness of consensus clustering. We then used Kaplan--Meier (KM) analysis to investigate CRGs associated with prognosis. Gene Set Variation Analysis (GSVA) is an unsupervised and non-parametric method that calculates gene set enrichment scores for each sample [13]. We conducted pathway enrichment analysis using GSVA and analyzed the differences in biological functions.

### 2.3. Consensus Clustering Analysis of Differentially Expressed Genes (DEG) between the Two CRGclusters

We used the R package limma to identify DEGs between two CRG clusters, setting a threshold of |log2-fold change (FC)| ≥ 0.585 and an adjusted *p*-value < 0.05. To perform enrichment analysis on the DEGs, we used the R package clusterProfiler to conduct Gene Ontology (GO) and Kyoto Encyclopedia of Genes and Genomes (KEGG) analysis. We then used univariate Cox regression analysis to identify DEGs associated with prognosis and screened for significant DEGs (*p*-value < 0.05) for subsequent consensus clustering (geneCluster) analysis. Finally, we used KM analysis to determine different geneClusters associated with prognosis.

### 2.4. Establishment of Risk Score Model

The merged cohort was randomly divided into a training and a test group in a 1:1 ratio. In the training group, we used the least absolute shrinkage and selector operation (LASSO) Cox regression to narrow down the list of screened prognostic genes and reduce the risk of overfitting. Multivariate Cox regression was then used to identify the genes suitable for constructing a risk model and to calculate their coefficients. The prognostic risk score was determined using the following formula:

$$Riskscore=\overset{n}{\underset{i=1}{\sum }}X_{i}\times \beta_{i}$$

where *n, X_i_*, and *β_i_* represent the total number, FPKM value, and the corresponding regression coefficient of genes. The median risk score in the training group was used as the cutoff value to divide the cohort into high- and low-risk groups, which was also applied to the test group. KM analysis was performed to compare the OS between the two groups, while time-dependent ROC analysis was used to assess the sensitivity and specificity of the risk model. Finally, a nomogram was constructed by integrating the risk scores with other clinicopathological features of LUAD patients. We utilized calibration curve analysis to validate the clinical applicability of the nomogram. Additionally, we investigated prognostic differences between subgroups stratified by age, gender, clinical stage, CRGcluster, and geneCluster. Single sample gene set enrichment analysis (ssGSEA) is a method for evaluating the relative activity or enrichment of predefined sets of genes in a single biological sample. We employed ssGSEA to explore expression differences in immune-related functional gene sets between different clusters. The associations among CRGcluster, geneCluster, risk score, and survival status were plotted using packages ggalluvial.

### 2.5. The Association between Risk Score and Tumor Mutation Burden (TMB)

We analyzed the differences in TMB between the high- and low-risk groups. Correlation scatter plots and boxplots were generated to examine the impact of the risk score on TMB. Furthermore, we conducted KM analysis to investigate the association between TMB and prognosis.

### 2.6. Analysis of Risk Score in the Role of TME

The ESTIMATE algorithm employs ssGSEA to generate stromal, immune, and ESTIMATE scores for a given sample [14]. The immune score, stromal score, and ESTIMATE score were utilized to assess the correlation between the risk score and TME. The CIBERSORT algorithm is a useful approach for characterizing diverse cell types in high-throughput tumor gene expression datasets [15]. The fractions of 22 immune cells in each LUAD sample were determined through the CIBERSORT algorithm. In addition, the association between risk score and various immune checkpoint genes was investigated.

### 2.7. Assessment of Immunotherapy Response and Validation of Risk Model

To predict the sensitivity of immunotherapy, a comparison of immunophenotype scores (IPS) was performed between high-risk and low-risk groups using The Cancer Immunome Atlas (TCIA) database (https://tcia.at/, accessed on 18 June 2022) [16]. To validate the risk model for immunotherapy response prediction, the publicly available IMvigor210 cohort was collected and utilized (IMvigor210: http://research-pub.gene.com/IMvigor210CoreBiologies, accessed on 5 August 2022) [17]. Additionally, the Tumor Immune Dysfunction and Exclusion (TIDE) tool was also employed to forecast immunotherapy response (http://tide.dfci.harvard.edu/, accessed on 18 June 2022) [18].

### 2.8. Stemness Score Analysis (RNAss) and Prediction of Chemotherapy Sensitivity

The RNAss was correlated with the risk score through a correlation analysis and subsequently plotted. Using package “pRRophetic”, the inhibitory concentration (IC50) of several key chemotherapeutic agents, including cisplatin, paclitaxel, docetaxel, gemcitabine, vinorelbine, elesclomol, and metformin, were compared in high- and low-risk groups.

### 2.9. Cell Culture

The A549 cell line was obtained from the Chinese Academy of Science Cell Bank (Shanghai, China) and cultured in RPM-1640 medium (Gibco, #31870074) supplemented with 10% FBS (Gibco, #16140071), 100 U/mL penicillin, and 100 U/mL streptomycin (Gibco, #15070063) at 37 °C in a humidified incubator (ThermoFisher, Waltham, USA, #3311) with 5% CO2. To knock down DLGAP5 expression, Lipofectamine^®^ 2000 reagent (ThermoFisher, #11668019) was used to transfect A549 cells with 50 nM of small interfering (si) RNA targeting DLGAP5, which was designed by Sangon Biotech (Shanghai, China) following the protocols. The sequences of siRNA targeting DLGAP5 were shown in Table S1.

### 2.10. Cell Counting Kit-8 (CCK-8) Assay

The cell viability of the A549 cell was monitored by CCK-8 (Biosharp, #BS350B). We seeded the A549 cell (3 × 10^3^/well) in the 96-well plates and determined the optical density (OD_450_) at 0, 24, 48, 72, and 96 h, respectively.

### 2.11. Statistical Analysis

The R software (version 4.1.2) was used for all statistical analysis and data visualizations. The student’s *t*-test, Wilcoxon rank sum test, and log-rank test were used for statistical comparisons, with *p* < 0.05 considered a statistical difference. Image processing was performed using Adobe Illustrator (CC 2017).

## 3. Results

### 3.1. The Landscape of 12 CRGs in LUAD

**Figure 2:**
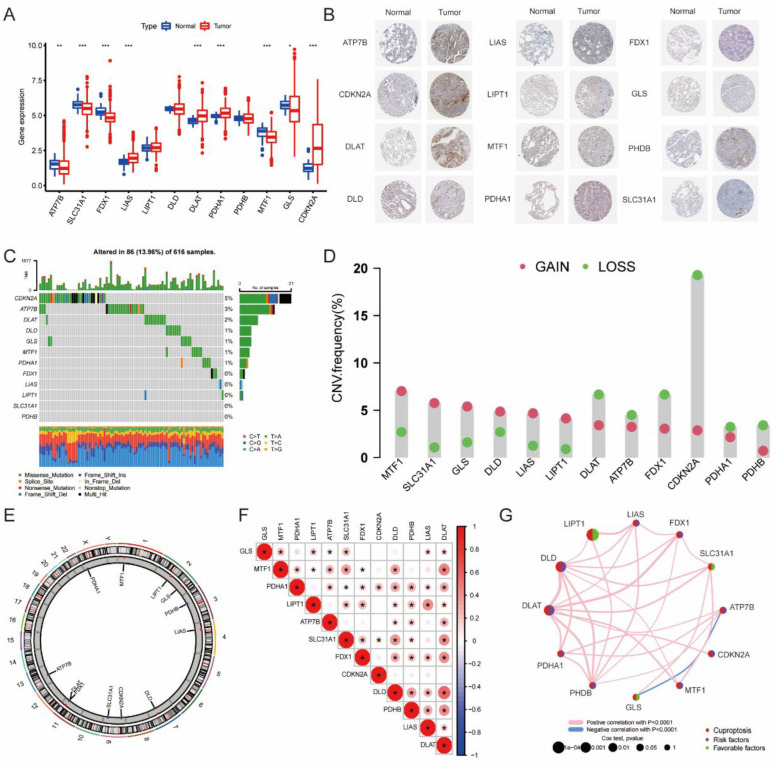
The landscape of CRGs in LUAD. (**A**) Cuproptosis-related gene (CRG) expression in LUAD tumor tissues and controls. (**B**) IHC staining of CRG in LUAD tumor tissues and adjacent tissues. (**C**) The mutation frequency of CRGs in LUAD. (**D**) The CNV of CRGs in LUAD. (**E**) CNV locations of CRGs on the chromosome. (**F**,**G**) CRG correlation analysis and regulatory network. * *p* < 0.05, ** *p* < 0.01, *** *p* < 0.001 indicate that the difference is statistically significant.

The mRNA levels of 12 CRGs were analyzed in both LUAD tumors and adjacent normal tissues. As depicted in Figure 2A, the results revealed that LIAS, DLAT, PDHA1, and CDKN2A were significantly upregulated in LUAD tumors compared to normal tissues, while ATP7B, SLC31A1, FDX1, MTF1, and GLS were significantly downregulated in tumor tissues. We then evaluated the IHC staining of CRGs and found a substantially stronger IHC signal in LUAD tumor tissues (Figure 2B). Among the 12 CRGs, their mutation frequency was relatively low in LUAD, with CDKN2A exhibiting the highest mutation rate (5%), followed by ATP7B (3%) and DLAT (2%). FDX1, LIAS, LIPTQ, SLC31A1, and PDHB showed no mutation in LUAD tumor tissues (Figure 2C). The CNV analysis revealed that CDKN2A, DLAT, FDX1, PDHB, ATP7B, and PDHA1 had a high frequency of CNV loss, while other genes exhibited a widespread frequency of CNV gain (Figure 2D). Figure 2E exhibits the detailed locations of these CNV alterations on the respective chromosomes. We further explored the correlation between the expression of CRGs depicted in Figure 2F,G, and nearly all CRGs exhibited strong positive associations with each other, except GLS negatively correlated with ATP7B. The heterogeneous expression pattern of CRGs in LUAD patients suggests that they may play a critical role in the development and progression of the disease.

### 3.2. Identification of CRGcluster in LUAD

**Figure 3:**
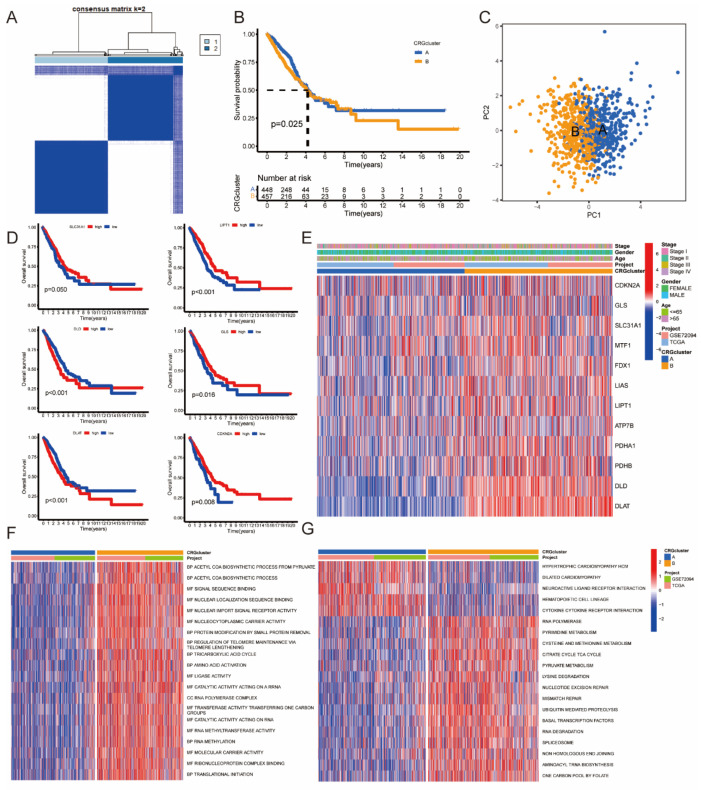
The CRGcluster in LUAD. (**A**) Clustering of patients with LUAD based on 12 CRG expression. Consensus clustering matrix for k = 2. (**B**) Survival analysis of different CRGclusters. (**C**) PCA analysis of different CRGclusters. (**D**) Survival analysis of prognostic CRGs. (**E**) The heatmap of different CRGs in CRGclusters and association with clinicopathological variables. (**F**,**G**) Functional and biological differences between different CRGclusters.

The unsupervised clustering analysis of the expression profiles of 12 CRGs using the consensus matrix k = 2 divided the entire cohort into two distinct clusters, namely A (*n* = 473) and B (*n* = 485) (Figure 3A). KM survival analysis of the two clusters suggested a poor OS in Cluster B (Figure 3B). PCA analysis reflected that CRGcluster could accurately distinguish LUAD patients, and this was also confirmed by the heatmaps (Figure 3C,E). Furthermore, the differential expressions of LIPT1, DLD, GLS, SLC31A1, DLAT, and CDKN2A were associated with prognosis in LUAD (Figure 3D). The GSVA analysis revealed differences in the top 20 most significant pathways between CRGcluster A and CRGcluster B, including the acetyl-CoA biosynthetic process, TCA cycle, and pathways associated with cuproptosis (Figure 3F–G). This might indicate a different cuproptosis status in CRGcluster A and CRGcluster B.

### 3.3. Identification of GeneCluster in LUAD

**Figure 4:**
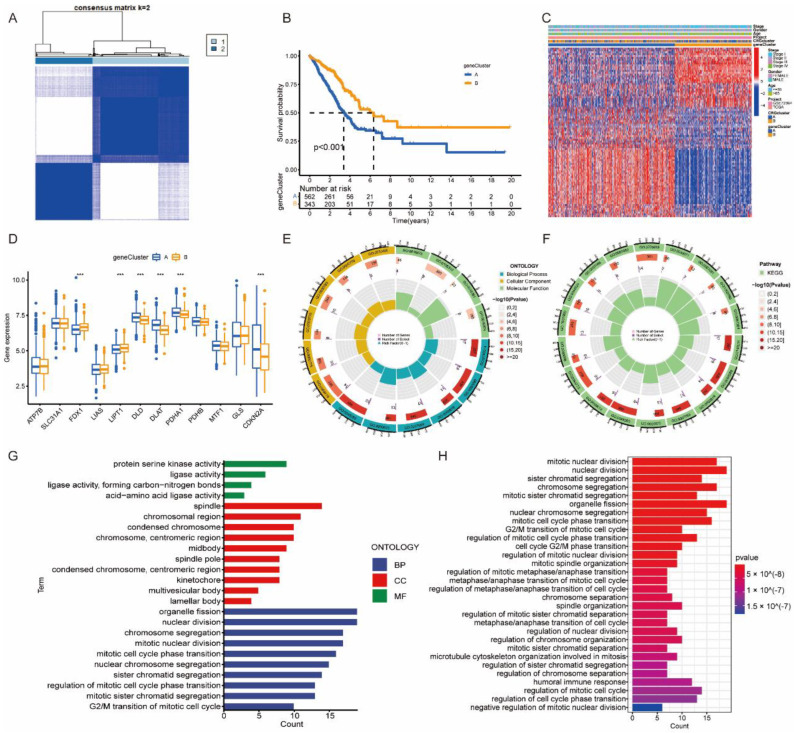
The geneCluster in LUAD. (**A**) Clustering of patients with LUAD based on 91 DEG expressions. Consensus clustering matrix for k = 2. (**B**) Survival analysis of different geneClusters. (**C**) The heatmap of DEGs in geneClusters and association with clinicopathological variables. (**D**) CRG expressions in different geneClusters. (**E**–**H**) GO and KEGG analysis of biological functions of 91 DEGs. *** *p* < 0.001 indicate that the difference is statistically significant.

Differential expression analysis identified 107 DEGs between CRGcluster A and CRGcluster B. We conducted a uni-Cox regression analysis to identify 91 genes that exhibited significant prognostic value (*p* < 0.05) (Table S1). Subsequently, a second consensus clustering based on the 91 DEGs was performed, aiming to separate the merged cohort into different gene signature subtypes. Two geneClusters (A and B) were found (Figure 4A). KM analysis suggested geneCluster B had a better prognosis compared with A (*p* < 0.001) (Figure 4B). The association among CRGclusters, geneClusters, and clinicopathological variables was investigated and shown in Figure 4C. Furthermore, the mRNA expressions of FDX1 and LIPT1 were higher in geneCluster B, while the DLD, DLAT, PDHA1, and CDKN2A were higher in geneCluster A, with significant differences (*p* < 0.001) (Figure 4D). In addition, functional enrichments using GO and KEGG pathway analysis were conducted using the 91 DEGs. GO results showed that these genes were involved in the protein serine kinase activity and ligase activity (Figure 4E,G). KEGG results demonstrated the pathways were associated with mitotic nuclear division and organelle fission (Figure 4F,H).

### 3.4. Construction of Risk Score Model Based on 91 DEGs

**Figure 5:**
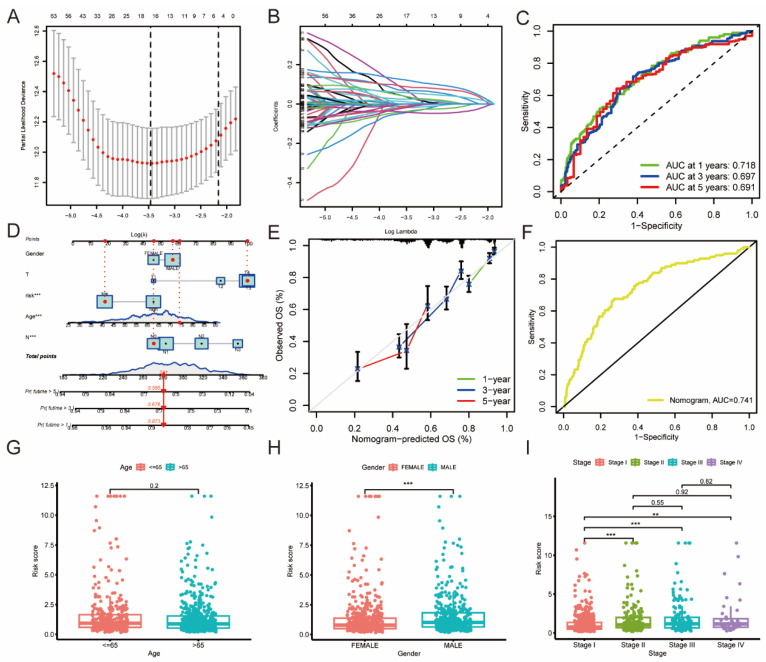
Construction of the risk model in LUAD. (**A**,**B**) LASSO regression analysis with a 10-fold cross-validation for the prognostic value of the 91 DEGs. (**C**) The AUC values of risk model at 1, 3, and 5 years. (**D**) The nomogram using clinicopathological variables. (**E**) Calibration curve for the overall survival (OS) of the nomogram. (**F**) The AUC values of nomogram at 1 year. (**G**–**I**) The risk score distributions in age, gender, and stage variables. ** *p* < 0.01, *** *p* < 0.001 indicate that the difference is statistically significant.

We found no statistically significant differences between the training and test groups in terms of their baseline characteristics (Table 1). Lasso and multivariate Cox analysis yielded 11 prognostic genes in the training cohort, including LTB, DLGAP5, TPPP3, FAM83A, ABCC2, VSIG2, CPS1, CYP4B1, CLDN6, FGB, and KRT6A (Figure 5A,B).

Table 2 displays the regression risk coefficients for each gene. KM survival analysis revealed that patients in the low-risk group had a significantly better prognosis compared to those in the high-risk group in the training cohort (*p* < 0.001) (Figure S1A). The heatmap results indicated that LTB, TPPP3, VS2G2, and CYP4B1 were upregulated in the low-risk group, while others were upregulated in the high-risk group (Figure S1D). Additionally, the distribution of the risk score is illustrated in Figure S1G–J, where survival time and living status are presented. Using the same median cutoff value, similar results were observed in the test cohort (Figure S1B,E,H,K) and total cohort (Figure S1C,F,I,L). The AUC values of the risk model at 1, 3, and 5 years were 0.718, 0.697, and 0.691, respectively, demonstrating a good prediction performance (Figure 5C). This confirmed that the risk model might be used as a novel method to stratify LUAD patients and predict patients’ prognoses. Moreover, the clinicopathologic variables were then combined with the risk model to construct a nomogram to further optimize the prediction performance (Figure 5D). As shown in Figure 5E, the slopes of the correction curve were close to 1, indicating good prediction accuracy. The AUC value of the nomogram was 0.741, better than the risk model alone (Figure 5F). We then investigated the risk score distributions in different clinicopathological groups. Male and late-stage LUAD patients tended to have a higher risk score, with *p* < 0.05 (Figure 5G–I).

### 3.5. The Association between Risk Score and Different Clusters

**Figure 6:**
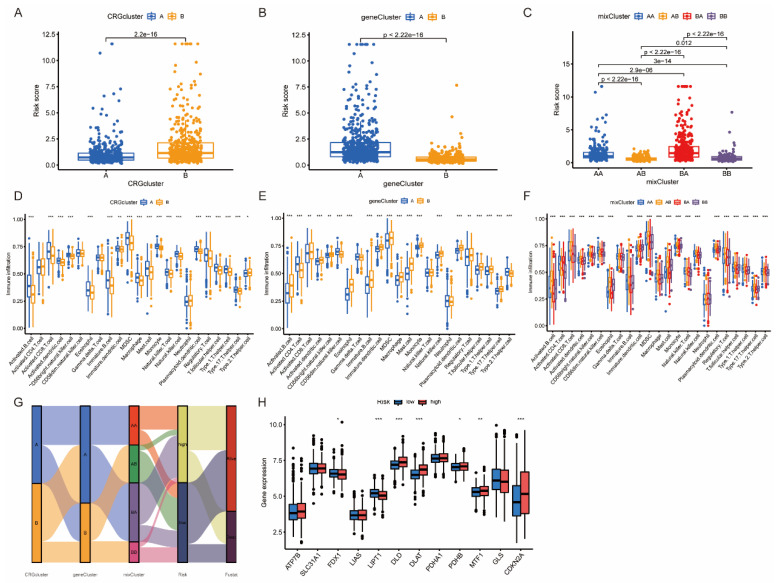
The detailed analysis of CRGcluster, geneCluster, and mixCluster. (**A**–**C**) The risk score distributions in CRGclusters, geneClusters, and mixClusters. (**D**–**F**) Immune cell infiltrations in different CRGclusters, geneClusters, and mixClusters. (**G**) Sankey diagram depicting the connections among risk score, different clusters, and survival status. (**H**) CRG expression in different risk groups. * *p* < 0.05, ** *p* < 0.01, *** *p* < 0.001 indicate that the difference is statistically significant.

The risk score was compared across different CRGclusters and geneClusters. The results showed that CRGcluster B and geneCluster A had higher risk scores (Figure 6A,B). We then defined the mixCluster by combing the CRG- and geneClusters results (Figure 6C). AB subgroup exhibited the lowest risk scores, while the BA subgroup exhibited the highest risk scores, with *p* < 0.05. The immune infiltration score was evaluated in different CRGclusters, geneClusters, and mixClusters. The analysis revealed that there were significant increases in activated B cells, activated CD8+ T cells, and activated dendritic cells in CRGcluster A, geneCluster B, and mixCluster AB, respectively (Figure 6D–F). The above results demonstrated that the immune infiltration status of CRGclusters and geneClusters were totally different. The combined mixCluster might more accurately reflect the immune status of the LUAD merged cohort. The Sankey diagram showed the connections of LUAD patients in CRGclusters, geneClusters, mixClusters, different risk groups, and survival status (Figure 6G). The risk score showed a positive correlation with the expression of DLD, DLAT, PDHB, MTF1, and CDKN2A, while it showed a negative correlation with the expression of FDX1 and LIPT1 (Figure 6H).

### 3.6. The Association between Risk Score and TMB

**Figure 7:**
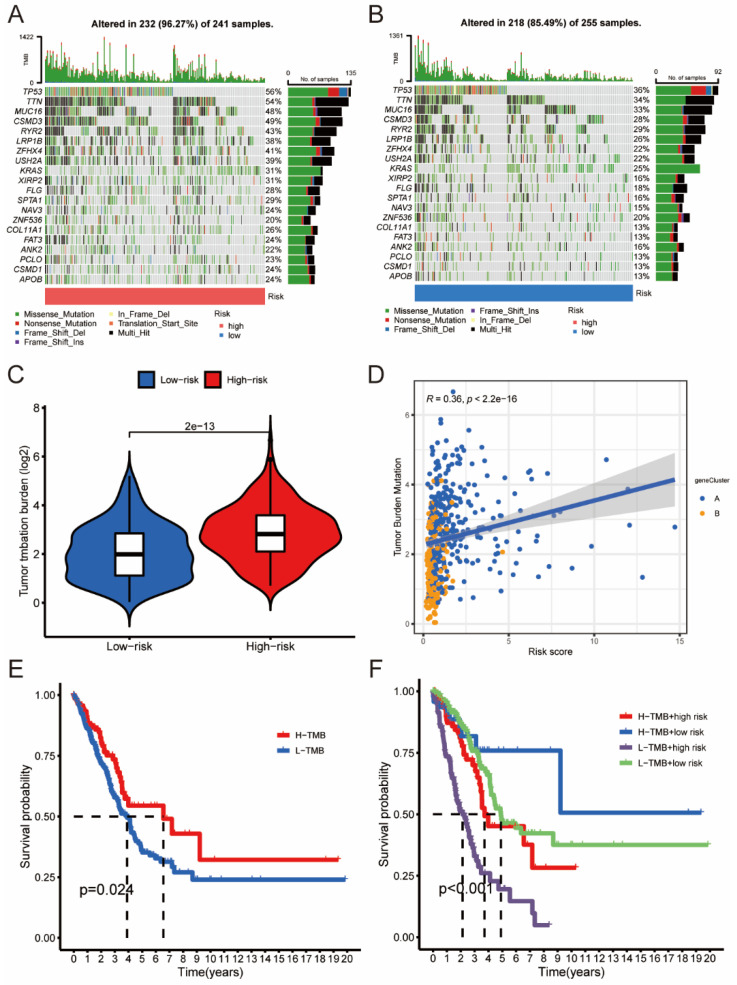
Correlations between the risk score and somatic variants. (**A**,**B**) The mutation rates of top 15 genes in the high- and low-risk groups. (**C**) Tumor mutation burden between high- and low-risk groups. (**D**) Correlation among TMB, geneClusters, and risk score in LUAD patients. (**E**,**F**) Comprehensive survival analysis based on risk score and TMB.

We plotted the distribution of the top 20 gene mutations in the different risk groups and calculated the TMB (Figure 7A,B). The combined mutation frequency of the top 20 genes was 96.27% and 85.49% in the high-risk and low-risk groups, respectively. Notably, the TMB of commonly mutated genes in LUAD, such as TP53 and TTN, was 56% and 54% in the high-risk group, whereas, in the low-risk group, it was 36% and 34%, respectively (Figure 7A,B). Additionally, the TMB was significantly higher in the high-risk group compared to the low-risk group (Figure 7C). The correlation between TMB and risk score was confirmed by correlation analysis, with a *p*-value < 0.001 (Figure 7D). Furthermore, as shown in Figure 7E, patients with high TMB had better overall survival (OS) than those with low TMB. Combining TMB and risk score resulted in a more accurate stratification of LUAD patients, as demonstrated in Figure 7F. Notably, the subgroup of low-risk patients with high TMB had the best prognosis.

### 3.7. The Association between Risk Score and TME

**Figure 8:**
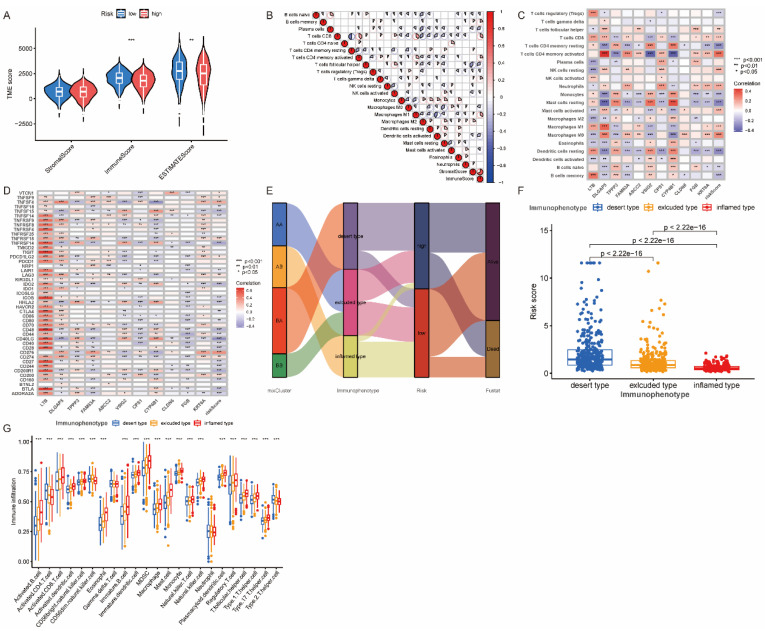
Correlation between the risk score and tumor microenvironment (TME) in the LUAD patients. (**A**) Comparison of immune scores, stromal scores, and ESTIMATE scores in different risk groups. (**B**) The correlation among different immune cells, immune scores, and stromal scores. (**C**) The correlation between immune cells and risk scores. (**D**) The correlation between immune checkpoints and risk scores. (**E**) The connection among mixClusters, immunophenotype, risk score, and survival status. (**F**) The risk score distribution in different immunophenotypes. (**G**) Immune cell infiltrations in different immunophenotypes. * *p* < 0.05, ** *p* < 0.01, *** *p* < 0.001 indicate that the difference is statistically significant.

The low-risk group displayed significantly higher immuneScore and ESTIMATEScore than the high-risk group, as shown in Figure 8A (*p* < 0.001), suggesting a stronger antitumor immune response in the former. The positive correlation between immuneScore and CD8 T cells, activated CD4+ memory T cells, and M1 macrophages, as well as the positive correlation between stromalScore and M2 macrophages and the negative correlation with T helper cells, is illustrated in Figure 8B. Figure 8C shows that risk scores were positively correlated with M0 macrophages, neutrophils, and activated CD4+ memory T cells. Most immune checkpoint genes were negatively correlated with risk scores, while LTB displayed a strong and positive correlation with almost all immune checkpoint genes, as shown in Figure 8D.

Considering the immune infiltration status, mixCluster AB was defined as the inflamed immunophenotype, mixCluster BA as the desert immunophenotype, and mixCluster AA and BB as excluded immunophenotype (Figure 8E). The inflamed immunophenotype exhibited the lowest risk scores and highest immune infiltration, while the desert immunophenotype exhibited the highest risk scores and lowest immune infiltration (Figure 8F,G).

### 3.8. The Association between Risk Score and Immunotherapy Response

**Figure 9:**
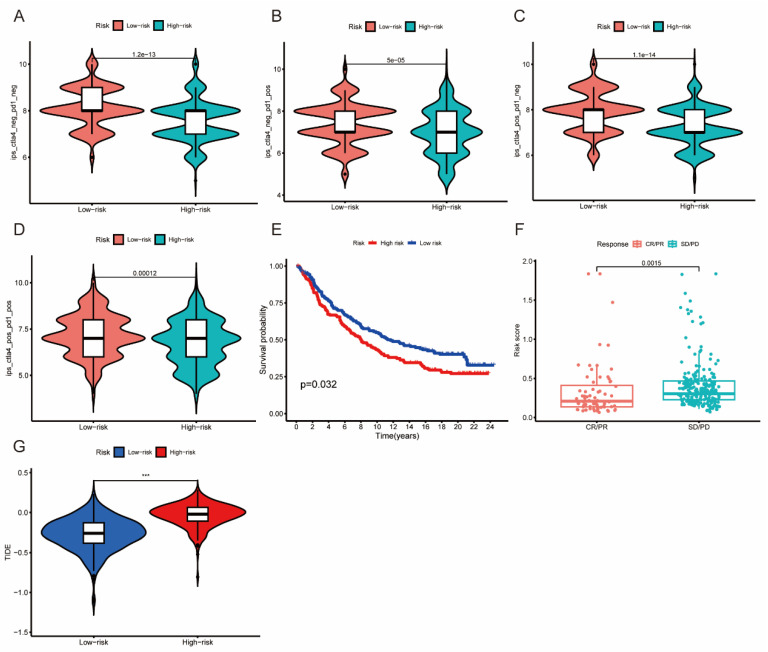
(**A**–**D**) The immunotherapy response between high- and low-risk groups using TCIA database; (**E**) Survival analysis of IMvigor210 cohort using our risk model; (**F**) Risk score distributions of CR/PR and SD/PD subgroup in the IMvigor210 cohort; (**G**) The TIDE scores between high- and low-risk groups. *** *p* < 0.001 indicate that the difference is statistically significant.

To further demonstrate the association between the risk score and response to immunotherapy, we examined the IPS of LUAD patients using the TCIA database. Patients in the low-risk group showed higher IPS values across all subgroups, indicating a more favorable response to immunotherapy (Figure 9A–D). To validate the predictive ability of our risk model for immunotherapy response, we retrieved the IMvigor210 cohort from a previous study and stratified patients into high- and low-risk groups using our predefined cutoff value. The low-risk group had a significantly better prognosis (*p* < 0.05) and showed higher rates of complete response (CR) and partial response (PR) (*p* < 0.01) compared with the high-risk group (Figure 9E,F). Additionally, the low-risk group exhibited lower TIDE scores than the high-risk group, suggesting a reduced potential for immune escape and a better response to immunotherapy (Figure 9G).

### 3.9. RNAss Analysis and Prediction of Chemotherapy Sensitivity

**Figure 10:**
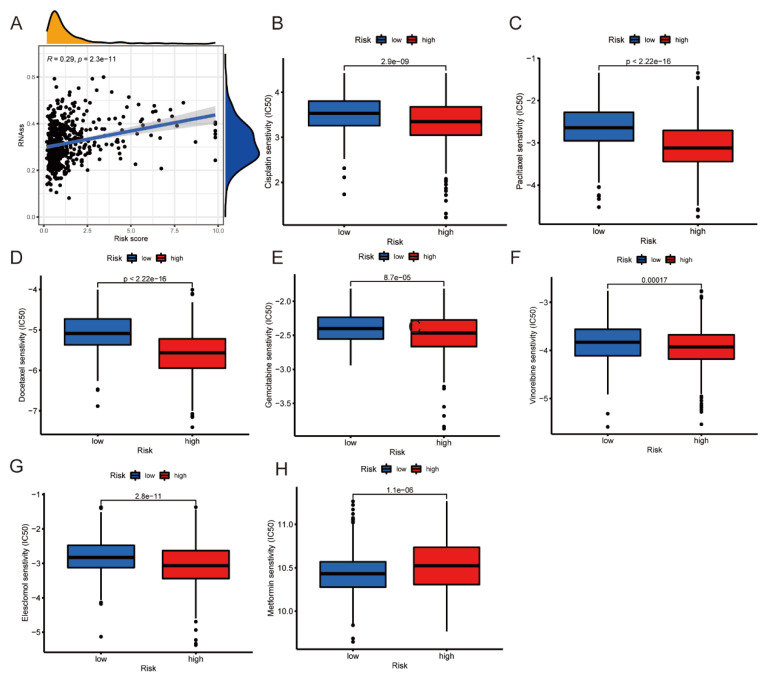
(**A**) The correlation of RNAss with the risk score. (**B**–**H**) The IC_50_ distribution difference of 7 commonly used drugs between high- and low-risk groups.

We then evaluated the association between risk score and RNAss. A positive correlation was identified between risk score and RNAss (Figure 10A). The pRRophetic algorithm was applied to assess the chemotherapy sensitivity of different antitumor drugs. The high-risk group was accompanied by a lower IC50 in cisplatin, paclitaxel, docetaxel, gemcitabine, vinorelbine, and elesclomol, reflecting a better antitumor efficacy (Figure 10B–G), while the low-risk group was accompanied with a lower IC50 in metformin, indicating a better drug sensitivity (Figure 10H).

### 3.10. Knockdown of DLGAP5 Inhibits the Proliferation of A549 Cell Line

**Figure 11:**
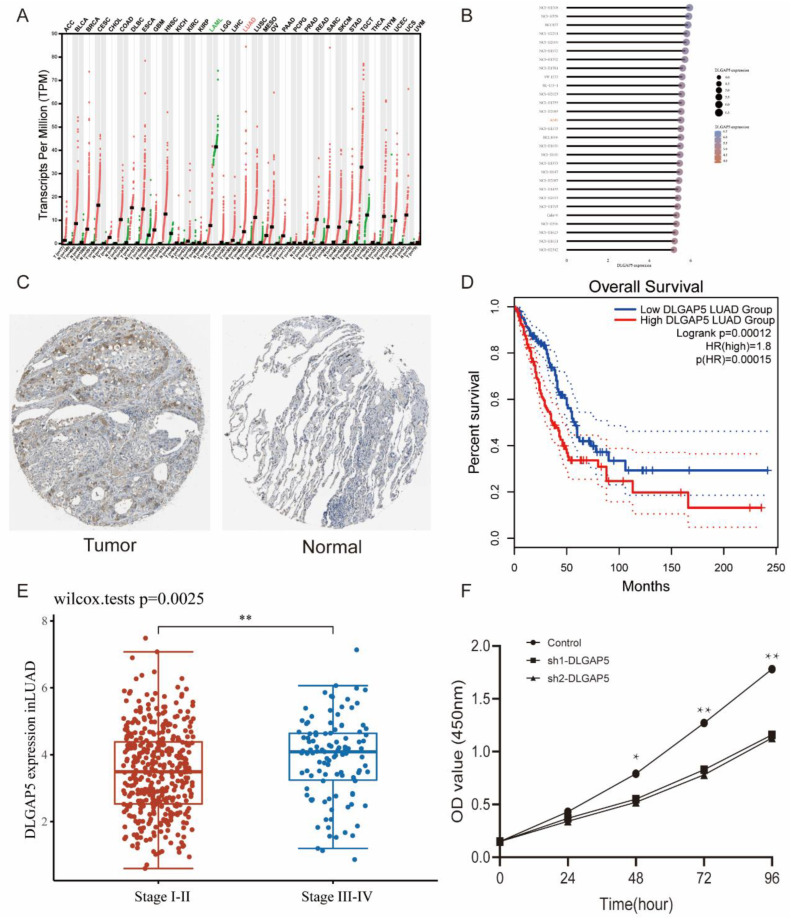
(**A**) Pan-cancer analysis of DLGAP5 in different tumors. (**B**) The expression of DLGAP5 in different LUAD cell lines. (**C**) IHC staining of DLGAP5 in LUAD tumor tissue and adjacent tissue. (**D**) Association of DLGAP5 expression with OS of LUAD patients. (**E**) Relative expression of DLGAP5 in early and late stage LUAD patients. (**F**) The effect of DLGAP5 knockdown on the proliferation of A549 cell line. Experiments were repeated three times. * *p* < 0.05, ** *p* < 0.01, indicate that the difference is statistically significant.

Because the HR value of DLGAP5 was the most significant among the 11 model genes, DLGAP5 was chosen for further validation. The pan-cancer analysis demonstrated that the mRNA expressions of DLGAP5 were higher in nearly all tumors compared with normal controls, except LAML (Figure 11A). Cancer cell line database (CCLE) results revealed that the expression of DLGAP5 in the A549 cell line was at the middle level among all cell lines and was chosen for CCK-8 experiments (Figure 11B). Substantial stronger expression of DLGAP5 protein was found in LUAD tumor tissues compared with normal tissues through IHC staining from the HPA database (Figure 11C). Furthermore, KM analysis confirmed that patients with high DLGAP5 expression had a poorer prognosis than those with low DLGAP5 expression (Figure 11D). The expression levels of DLGAP5 in stage III-IV LUAD patients were significantly higher than those in stage I-II LUAD patients (Figure 11E). In vitro experiments demonstrated that the knockdown of DLGAP5 inhibited the proliferation of the A549 cell line (Figure 11F). In summary, DLGAP5 increases the tumorigenicity of the LUAD A549 cell line.

## 4. Discussion

In this study, we developed a risk model that can assess immunophenotype and predict response to immunotherapy. Our model comprised 11 genes, many of which have been previously identified. For example, TPPP3 knockdown has been shown to inhibit tumor growth in vitro and in vivo and induce apoptosis and cell cycle arrest in lung cancer [19]. FAM83A overexpression has been linked to increased cell proliferation, migration, and epithelial-mesenchymal transition (EMT) in A549 and H1299 cell lines [20]. Silencing CPS1 in KRAS/LKB1-mutant lung cancer cells has been found to lead to pyrimidine depletion, S-phase progression, DNA-polymerase stalling, DNA damage, and cell death [21]. CLDN6 has been shown to regulate several signaling pathways that promote lung cancer malignant phenotypes, such as proliferation, migration, invasion, and drug resistance [22]. The high expression of KRT6A has been associated with the promotion of A549 cell line growth and invasion through increasing MYC-mediated G6PD expression [23]. Further analysis of these genes and their interaction with cuproptosis may lead to the development of novel treatment strategies for LUAD.

Based on the abundance of tumor-infiltrating lymphocytes (TILs), TME can be classified into hot tumors (inflamed type) and cold tumors (desert and excluded type) [24]. Hot tumors generally exhibit richer immune infiltrations and better immunotherapy response than cold tumors [25,26,27], while poor TILs infiltrations are associated with immunotherapy resistance [28,29]. Thus, accurately distinguishing cold tumors from hot tumors can improve immunotherapy efficiency and reduce unnecessary medical costs. Remodeling TME and converting “desert” to “inflamed” may be potential ways to improve the prognosis of certain cold tumors. In this study, we defined immunophenotype based on the CRGcluster and geneCluster results and investigated the association between immunophenotype and risk score. The low-risk group was more likely to exhibit an inflamed immunophenotype, while the high-risk group was associated with a desert phenotype. External validation using the IMvigor210 cohort further confirmed this result. However, a larger cohort is needed to further demonstrate the connection between these two factors and confirm the authenticity and reliability of the risk model.

To date, several indicators have been applied for predicting immunotherapy response, including PD-L1, MSI-H, dMMR, and TMB [30]. However, none of these biomarkers have exhibited durable and reliable performance in stratifying patients suitable for immunotherapy. For example, the recent KEYNOTE-189 study demonstrated that patients with low PD-L1 expression could also benefit from immunotherapy [31]. Moreover, the latest results from the KEYNOTE-091 study showed that pembrolizumab could improve the time of disease-free survival in NSCLC patients regardless of PD-L1 expression [32]. In the present study, we found that low-risk patients presented with an inflamed phenotype TME, better response to immunotherapy, and low TIDE scores. External validation using the IMvigor210 cohort confirmed our results from different aspects. Thus, the risk model may serve as an effective indicator for prognosis, contribute to patient stratification, and fill the gap in terms of predicting immunotherapy response.

The CIBERSORT algorithm is a valuable tool for identifying various cell types in large-scale tumor gene expression datasets [15]. It works by using a specialized gene expression signature matrix to deconvolute the cell types of interest. Unlike previous methods, CIBERSORT employs a machine learning approach called support vector regression (SVR), which combines feature selection and robust mathematical optimization techniques to improve deconvolution performance [33]. Benchmarking experiments have shown that CIBERSORT is more accurate than other methods at identifying closely related cell subsets and mixtures containing unknown cell types in solid tissues.

In recent years, a number of studies have investigated the role of CRGs and cuproptosis-related lncRNAs (CRlncRNAs) in tumors through integrated analysis of TCGA and GEO databases [34,35]. LncRNAs, a type of non-coding RNA with a molecular weight of over 200 nucleotides, play a key role in regulating various pathophysiological processes [36,37,38]. Ma et al. developed a CRlncRNAs signature to predict the prognosis of LUAD patients [35]. Yang et al. applied a CRlncRNAs model to evaluate the immune infiltration status in head and neck squamous cell carcinoma [39]. Li et al. demonstrated the potential of CRlncRNAs in predicting immunotherapy response in LUAD [40]. These findings suggest that CRlncRNAs may serve as promising biomarkers for revealing the complex tumor microenvironment and aiding clinicians in formulating personalized immunotherapy for patients.

Several limitations of the present study need to be addressed. Firstly, the majority of our analyses were based on bioinformatics, and therefore, validation with real-world LUAD samples is needed to confirm the relationship between the risk model and immunophenotype. Secondly, cuproptosis is a newly proposed concept, and CRGs are still being discovered, so the genes used in this study may not be comprehensive. Thirdly, more in-depth validation experiments in vivo and in vitro are required to investigate the connection between cuproptosis, tumor immune functions, and immunotherapy response.

## 5. Conclusions

In summary, cuproptosis depicted novel molecular patterns in LUAD, including CRGcluster, geneCluster, and mixCluster. The risk model might reveal the different immunophenotypes and have a potential role in predicting immunotherapy response.

## Figures and Tables

**Table 1:** Baseline characteristics of the merged LUAD patients.

Characteristics	Training Cohort	Testing Cohort	Total	*p*-Value
Age				
≤65	172	180	352	0.6698
>65	268	262	530	
Gender				
Female	247	237	484	0.4944
Male	193	205	398	
Stage				
Stage I	250	269	519	0.6263
Stage II	96	89	185	
Stage III	71	66	137	
Stage IV	23	18	41	

LUAD: lung adenocarcinoma.

**Table 2:** The coefficient of model genes.

Model Gene ID	Coefficient	HR	HR.95 L	HR.95H	*p*
LTB	−0.137957879670175	0.417	0.233	0.748	0.003
DLGAP5	0.130544222141117	3.967	2.172	7.245	0.001
TPPP3	0.228761500541897	0.474	0.263	0.854	0.013
FAM83A	0.153655354188085	2.444	1.429	4.181	0.001
ABCC2	0.150266269384804	1.989	1.487	2.659	0.001
VSIG2	−0.0991593804465051	0.447	0.295	0.676	0.001
CPS1	0.0859641132043299	1.413	1.114	1.791	0.004
CYP4B1	−0.0839532602813634	0.616	0.464	0.818	0.001
CLDN6	0.0476999799180695	1.320	1.052	1.656	0.016
FGB	−0.0873497912904369	1.323	1.094	1.560	0.003
KRT6A	0.0866237588843813	1.567	1.254	1.958	0.001

## Data Availability

All data used in the study can be downloaded from the TCGA (https://portal.gdc.cancer.gov/, accessed on 18 June 2022) and GEO (https://www.ncbi.nlm.nih.gov/geo/, accessed on 18 June 2022, GSE72094). R code could be obtained from the corresponding author.

## Supplementary Materials

The following supporting information can be downloaded at: https://www.mdpi.com/article/10.3390/jpm13030482/s1, Figure S1: The correlation between the risk model and the prognosis of LUAD patients; Table S1: shRNA Sequence for DLGAP5 and Negative Control.
