# Supplementary material for: Cuproptosis Depicts Immunophenotype and Predicts Immunotherapy Response in Lung Adenocarcinoma
Source: J Pers Med. 2023 Mar 8;13(3):482. doi: 10.3390/jpm13030482 (PMC10051631; doi:10.3390/jpm13030482)
Supplement: Supplementary file 1 [file jpm-13-00482-s001.zip › Figure S1.zip!/Figure S1.pdf]

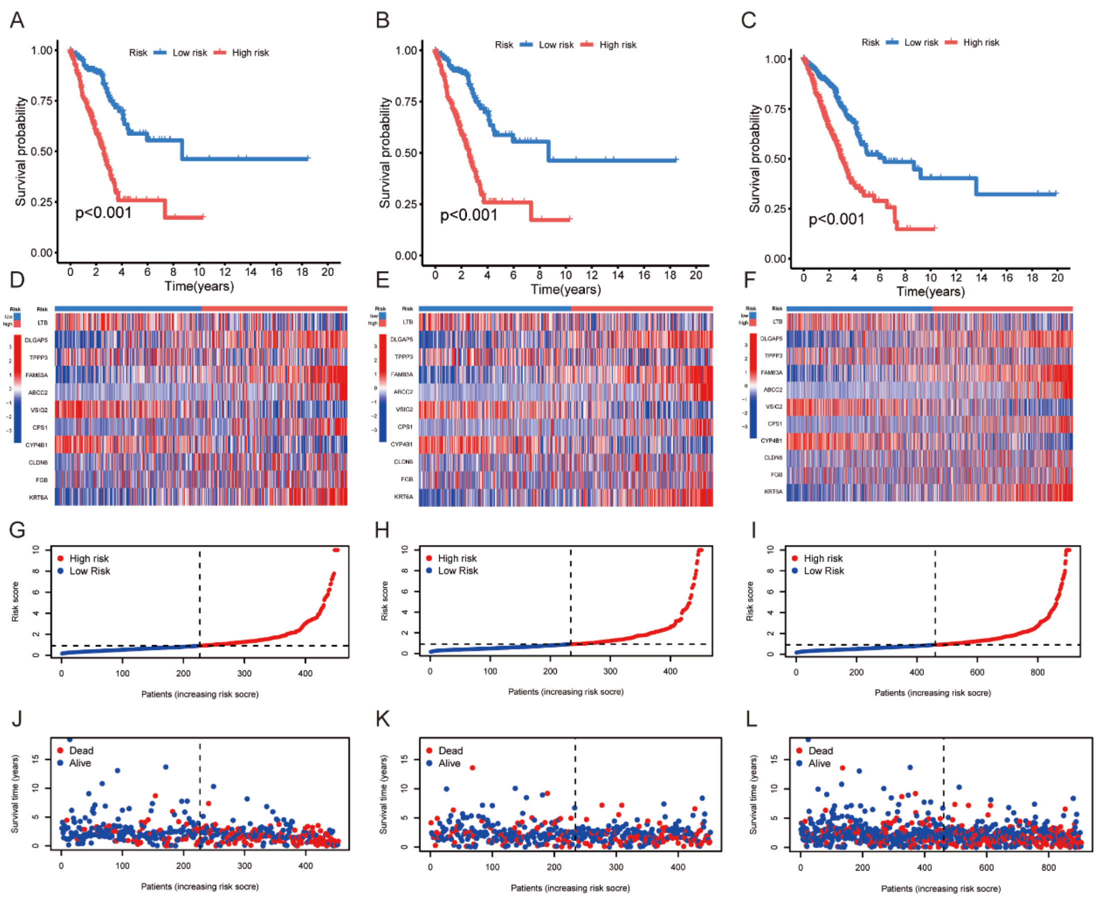

Figure S1: The correlation between the risk model and the prognosis of LUAD patients. (A, D, G&J) Training group; (B, E, H&K) Test group; (C, F, I&L) Total patients; (A-C) Kaplan-Meier analysis of the OS rate of LUAD patients. (D-F): Heat map showing the expression profiles of 11 model genes. (G-I): The distribution of the risk score among LUAD patients. (J-L): The number of dead and alive patients with different risk scores. Blue represents the number of survivors, and red represents the number of deaths.
