# Supplementary material for: Cuproptosis Depicts Immunophenotype and Predicts Immunotherapy Response in Lung Adenocarcinoma
Source: J Pers Med. 2023 Mar 8;13(3):482. doi: 10.3390/jpm13030482 (PMC10051631; doi:10.3390/jpm13030482)
Supplement: Supplementary file 1 [file jpm-13-00482-s001.zip › Supplementary material key packages and algorithms in the manuscript.zip!/Supplementary material key packages and algorithms in the manuscript.pdf]

**For ConsensusClusterPlus R package**: ConsensusClusterPlus provides a comprehensive approach to identify intrinsic groups of data in cancer research. Unsupervised class discovery is a useful technique for discovering these groups without prior knowledge. Consensus clustering (CC) is a popular method that provides both quantitative and visual evidence to estimate the number of unsupervised classes within a dataset. ConsensusClusterPlus is an R package that offers additional functionality and visualizations such as item tracking, item-consensus, and cluster-consensus plots. These new features provide users with detailed information that allows for more precise decisions in unsupervised class discovery compared with other methods.

**For Gene Set variation analysis (GSVA)**: Gene Set Variation Analysis (GSVA) is an unsupervised and non-parametric method that calculates gene set enrichment scores for each sample. GSVA determines enrichment scores by considering genes both inside and outside the gene set, similarly to a competitive gene set test. This approach allows estimation of gene set enrichment variation across samples. The methodology can be conceptualized as a transformation of the coordinate system of gene expression data, from genes to gene sets. This transformation enables the construction of pathway-centric models post-hoc, including differential pathway activity identification and survival prediction.

**For Single sample gene set enrichment analysis (ssGSEA)**: Single sample gene set enrichment analysis (ssGSEA) is a method for evaluating the relative activity or enrichment of predefined sets of genes in a single biological sample. The method involves ranking the expression values of all genes in the sample, calculating a score for each gene set based on the ranked expression values, and then normalizing the scores to obtain an ssGSEA score for each sample and each gene set. ssGSEA can be used to identify which biological pathways or gene sets are significantly activated or deactivated in a given sample, and it has been widely used in gene expression studies of cancer, immunology, and other fields.

**For CIBERSORT algorithm**: CIBERSORT algorithm employs a specialized knowledge base of gene expression signatures, called a "signature matrix," for the deconvolution of cell types of interest. Unlike previous efforts, CIBERSORT algorithm utilizes a machine learning approach called support vector regression (SVR), which combines feature selection and robust mathematical optimization techniques to improve deconvolution performance. Benchmarking experiments have demonstrated that CIBERSORT algorithm is more accurate than other methods in resolving closely related cell subsets and mixtures with unknown cell types in solid tissues. Therefore, CIBERSORT algorithm is a highly useful approach for characterizing diverse cell types in high-throughput tumor gene expression datasets. This paragraph has been added in the discussion section.

**For ESTIMATE algorithm**: The ESTIMATE algorithm employs ssGSEA to generate stromal, immune, and ESTIMATE scores for a given sample. In ssGSEA, gene expression values are rank-normalized and rank-ordered, and the empirical cumulative distribution functions of genes in the signature and remaining genes are calculated. A statistic is then derived by integrating the difference between the two empirical cumulative distribution functions, similar to gene set-enrichment analysis, but based on absolute expression rather than differential expression. The signatures related to stromal tissue and immune cell infiltration are used to define ssGSEA scores, which are combined to generate the ESTIMATE score.
