# Supplementary material for: Cuproptosis Depicts Immunophenotype and Predicts Immunotherapy Response in Lung Adenocarcinoma
Source: J Pers Med. 2023 Mar 8;13(3):482. doi: 10.3390/jpm13030482 (PMC10051631; doi:10.3390/jpm13030482)
Supplement: Supplementary file 1 [file jpm-13-00482-s001.zip › Table S1 shRNA.zip!/Table S1 shRNA.pdf]

Table S1 shRNA Sequence for DLGAP5 and Negative Control

Gene	shRNA
sh-DLGAP5-1	5'-ATTCAAACCACATCAGCCTGC-3'
sh-DLGAP5-2	5'-TTGACCGGAGAAGATAGAGGA-3'
sh-Control	5'-GAAACACAGCCTGTACGCCTG-3'
